# Biomechanical effect of metal augment and bone graft on cup stability for acetabular reconstruction of total hip arthroplasty in hip dysplasia: a finite element analysis

**DOI:** 10.1186/s12891-022-05168-1

**Published:** 2022-03-23

**Authors:** Yuzhu Wang, Mincong Wang, Chengguo Li, Yoshihiro Nakamura, Liwei Deng, Go Yamako, Etsuo Chosa, Chenglong Pan

**Affiliations:** 1 Department of Orthopaedic Surgery, The Fifth Affiliated Hospital of Southern Medical University, Guangzhou, Guangdong China; 2 Department of Orthopaedic Surgery, Faculty of Medicine, University of Miyazaki, Miyazaki, Miyazaki Japan; 3 Department of Radiology, The Fifth Affiliated Hospital of Southern Medical University, Guangzhou, Guangdong China; 4 Department of Mechanical Engineering, Faculty of Engineering, University of Miyazaki, Miyazaki, Miyazaki Japan

**Keywords:** Augment, Acetabular reconstruction, Cup stability, Finite element analysis

## Abstract

**Background:**

Different methods of acetabular reconstruction with total hip arthroplasty (THA) for Crowe II and III of adult developmental dysplasia of the hip (DDH) acetabular bone defect have been implemented clinically. However, the biomechanical effect of different augmented materials for acetabular reconstruction in THA on shell stability has never been discussed.

**Methods:**

In the present study, autologous bone graft (BG)and metal (Ti6Al4V) augment (MA) were simulated with several acetabular bone defect models of DDH in THA. The contact pressure and micromotion between the shell and host bone were measured for evaluating the shell stability using a finite element method.

**Results:**

The peak contact stress between shell and host bone was higher in the MA situation (12.45 vs 8.71 MPa). And the load transfer path was different, for BG models, the high local contact stresses were found at the junction of bone graft and host bone while for MA models the concentrated contact stresses were at the surface of MA. The peak relative micromotion between shell and host bone was higher in the MA situation (12.61 vs 11.13 µm). However, the peak micromotion decreased in the contact interface of MA and cup compared to the BG models.

**Conclusions:**

The higher micromotion was found in MA models, however, enough for bone ingrowth, and direct stronger fixation was achieved in the MA-cup interface. Thus, we recommended the MA can be used as an option, even for Crowe III, however, the decision should be made from clinical follow-up results.

## Introduction

Acetabular reconstruction with total hip arthroplasty (THA) for Crowe II and III of adult developmental dysplasia of the hip (DDH) is a challenge [1]. Because you have to premeditate the position of the arthroplasty cup, compared to Crowe I and IV, which the position of arthroplasty cup is the original true acetabular position in most cases, although acetabular reconstruction with THA for Crowe IV is much more difficult [2], the degree of acetabular bone defect [3], and the techniques of reconstruction [4, 5], In this study, augmentations for acetabular reconstruction of DDH with THA by restoring the original center of femoral head rotation in the situation of Crowe II and III bone defect were discussed [6].

Acetabular bone defect in DDH can be regarded as an inherent existence [7]. To what extent acetabular cup uncoverage affect the stability after THA matters. It has been suggested this uncoverage should not exceed 30% of the cup generally [8]. For obtain adequate bone coverage in the bone defect more than 30%, the use of a small cup size with medialization or high hip center positioning of cup for stable fixation of the acetabular component is an option [9]. However, the hip center of rotation (COR) was changed [10]. To restore the HCOR and establishing normal biomechanics of the hip, autologous bone graft was a traditional material for roof acetabular reconstruction, and the long-term outcomes was obtained [11], however, the complications such as the resorption of bone graft resulted in instability of acetabular component [12]. Recently, the metal augments were developed for acetabular reconstruction in primary and revision THA [13], and the short-term results was promising [14, 15]. Although the biomechanical behavior of different augment materials (Ti6Al4V vs Trabecular Metal) has been compared in stress level using the finite element method [16]. However, the biomechanical comparison of bone graft and metal augments on cup stability of the interface between acetabular component and host bone is missing, more information is needed. We hypothesized that the metal augment could provide a stable cup stability as equally as the bone graft provided.

The purpose of the study was to establish several acetabular bone defect models of DDH reconstructed with Ti6Al4V augment and autologous bone graft in THA and compare the influence of the two materials on biomechanical behavior (cup stability) of the interface between acetabular component and host bone using a finite element method.

## Materials and methods

### Construction of acetabular bone defect models of DDH

A healthy volunteer (Sex: male, age: 27 years, Height: 164 cm, Body weight: 66 kg) without any musculoskeletal disease or history of hip joint operations was recruited. quantitative computed tomography (QCT) was scanned in combination with a calibration phantom (B-MAS200, Kyoto-kagaku, Kyoto, Japan) for calibrating the bone mineral density [17]. The resolution of each CT image was 512 by 512 pixels with a slice thickness of 1.0 mm, and the pixel size was 0.782 mm/pixel under 120 kV and 102.50 mA conditions. We used the commercial software MIMICS (v 22, Materialise, Belgium) to reconstruct the intact right iliac and femoral bone, and methods can be found in our previous study [18].

**Fig. 1:**
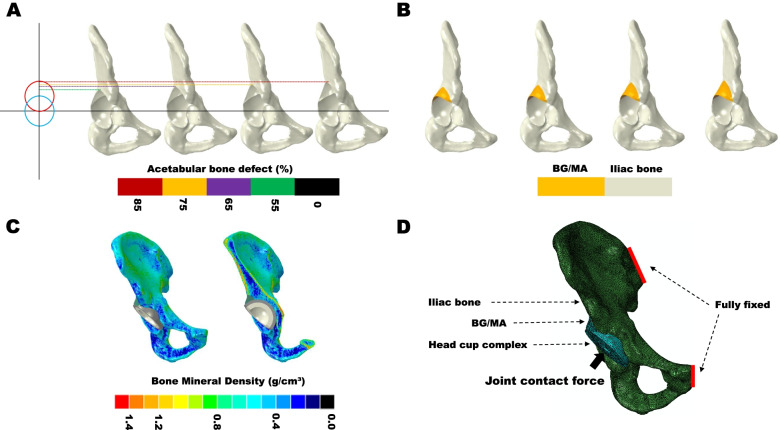
Finite element modeling of acetabular reconstruction of DDH in THA. **a** Acetabular bone defect modeling used in the study. **b** Acetabular reconstruction with BG/MA. **c** Iliac bone was assigned with heterogeneous material properties. **d** Loading and boundary conditions of finite element modeling

According to Crowe’s classification for adult developmental dysplasia of the hip (DDH) [19], there are two methods to evaluate the degree of DDH, one is subluxation of femoral head that can be measured by the elevation of femoral head center. Another one is the ratio of the distance of proximal dislocation to the pelvic height [20]. The acetabular bone defect models of Crowe II and III in THA were made by elevating the femoral head center or center of rotation of femoral head (COR) [21] from original COR (black solid line) to 55% (Green dotted line), 65% (purple dotted line), (Crowe II); 75% (yellow dotted line), 85% (red dotted line), (Crowe III) of the femoral head radius, which can be regarded as the COR dislocation percentage (Fig. 1a). When COR reached the top range of femoral head, the COR dislocation percentage was recorded as 100% [22].

### Reconstruction of acetabular bone defect models of DDH in THA

The acetabular bone defect models were simulated with Boolen operation using a CAD system (SolidWorks 2016, SolidWorks Corp, USA). The lost part of acetabular by Boolen operation was preserved as the metal augment (MA) (Ti6Al4V) and structural autologous bone graft (BG) geometrical shape (Fig. 1b) [23]. Two screws with the diameter of 6.5 mm were inserted to fix the MA or BG [16]. Acetabular cup (shell, linear) and femoral (head) prostheses were made in SolidWorks according to the size of the subject’s acetabulum and femoral head. The acetabular cup was a 52 mm PINNACLE cup without porous coating (Depuy, America) [24]. Cup inclination of 40 degrees and anteversion of 20 degrees were preset using anterior pelvic plane (APP) [25]. The ceramic liner (Depuy, America) with 32 mm femoral ceramic head (Link, Germany) were implanted [26]. The solid models were assembled for Reconstruction of acetabular bone defect models in THA.

### Material properties of finite element modeling

Mesh size of the models were approximately 1 mm with four-node tetrahedral elements, which has been validated from the study [27]. In this study, each element of iliac bone was assigned with isotropic heterogeneous Young’s modulus based on QCT data form our previous study [18] (Fig. 1c). the parameters used for converting Hounsfield Units (HU) to radiographic CT density (${\rho }_{QCT}(\mathrm{g}/{\mathrm{cm}}^{3})$ (Eq. (1)) were calculated from the B-MAS200 phantom [17], and from ${\rho }_{QCT}$ to Ash density (${\rho }_{ash}(\mathrm{g}/{\mathrm{cm}}^{3})$ (Eq. (2)) [28], then then the apparent density that was calculated from the ash density with a ratio of 0.6 [29] was converted to the elastic modulus (Eq. (3)) [30].

1 $${\rho }_{QCT}\left(g/{\mathrm{cm}}^{3}\right)=0.9863HU-2.0804$$

2 $${\rho }_{ash}(\mathrm{g}/{\mathrm{cm}}^{3})=0.877\times {\rho }_{QCT}+0.078$$

3 $$\mathrm{E}=6850{\rho }_{app}^{1.49}$$

Hip protheses (shell, linear, and head), two augmental materials and the screws were assigned with isotropic homogenneous elastic properties from literature (Table 1) [16, 22].

**Table 1:** Material properties of FE models used in the study

Components	Materials	Elastic modulus (MPa)	Poisson’s ratio
Iliac bone Bone graft	Bone	5.452–17,756.30 150	0.3
Acetabular shell	Tantalum	8963	0.31
Metal augment Screws	Titanium alloy	110,600	0.326
Liner Femoral head	Ceramics	350,000	0.22

### Loading and boundary conditions

Hip contact force of single-legged stance without taking account of muscles [24] was performed at the femoral head center (Fig. 1d). The pubic symphysis and sacroiliac joint were fully fixed to prevent translation and rotation. The interface of screws and bone was tied contact, the friction coefficient between bone and augment materials interface, the bone and cup interface were set as 0.8 [16, 22], which was press-fit contact pattern without screw implantation [31, 32], the head and linear interface was 0.06 [33]. The FE analysis was performed using a general-purpose FEA software program (ABAQUS 2019, Dassault Systems, Providence, RI).

### Evaluation of the simulation

The effect of different materials of augment on stability of acetabular cup was evaluated by the contact pressure with CPRESS [34, 35] and relative micromotion [21, 24, 36] in each of the DeLee and Charnley Zones [37]. The micromotion of the shell in the surrounding bone stock was evaluated using the relative tangential node displacements in the contact surface. The postprocessor ABAQUS enables the prediction of tangential displacements (CSLIP) in the two perpendicular directions t_1_ und t_2_ throughout the whole surface of the implant bed. The maximum amounts of micromotion were calculated in each finite element $n$ by calculation (Eq. (4)) [39]

4 $$Relative Micromotion (n)=\sqrt{{[CSLIP1(\mathrm{t}1, n)]}^{2}+{[CSLIP2(t2, n)]}^{2}}$$

## Results

**Fig. 2:**
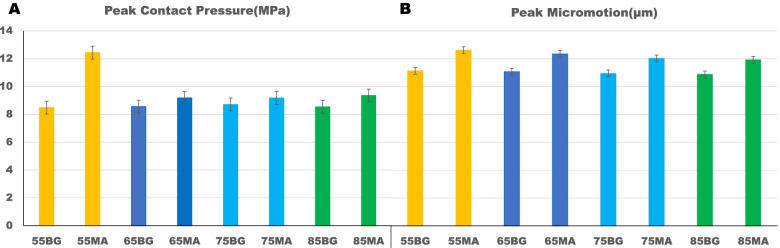
The peak values of prediction in cup-host bone interface (including cup-BG/MA interface) of different models. **a** The values of peak contact pressure **b** The values of peak micromotions

**Fig. 3:**
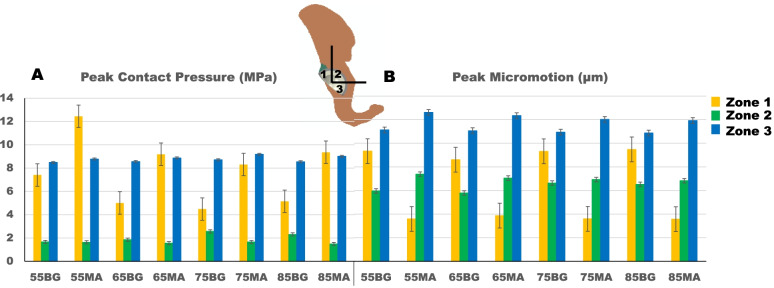
The peak values of prediction in each DeLee and Charnley zone of cup-host bone interface (including cup-BG/MA interface) of different models. **a** The values of peak contact pressure. **b** The values of peak micromotions

**Fig. 4:**
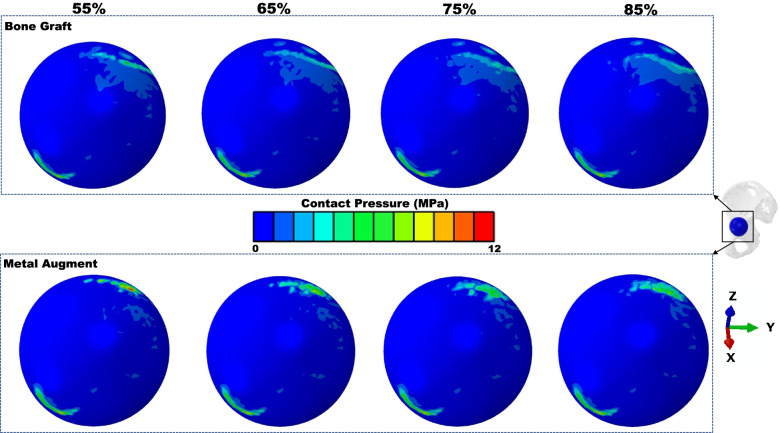
Contact stresses distribution of cup surface in different models

**Fig. 5:**
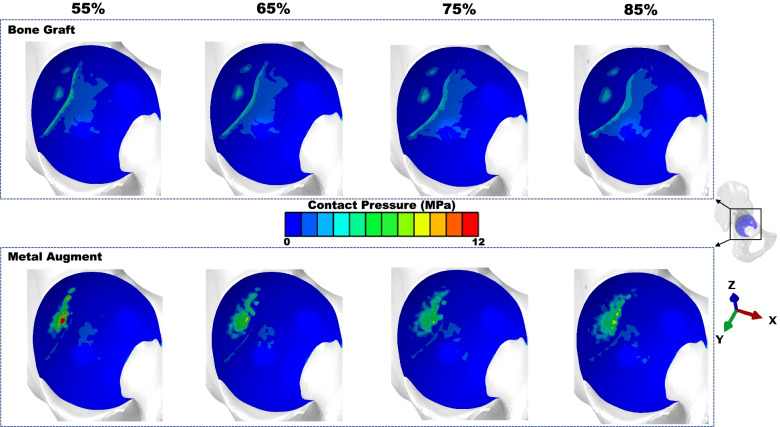
Contact stresses distribution of host bone (including BG/MA) surface in different models

The peak contact stress between shell and host bone (including BG/MA contact area) was higher in the MA situation (12.45 vs 8.71 MPa) (Fig. 2a). For BG situation, the higher contact stress was in zone 3 (8.71 MPa), for MA situation, the higher contact stress was in zone 1 (12.45 MPa) (Fig. 3a). The concentration of contact stress for the shell was present in the superoposterior corner of zone 1 and inferoanterior corner of zone 3 (Fig. 4), for the host bone (including BG/MA contact area), the concentration of contact stress was in the junction area of BG situation and over the MA surface of MA situation (Fig. 5).

**Fig. 6:**
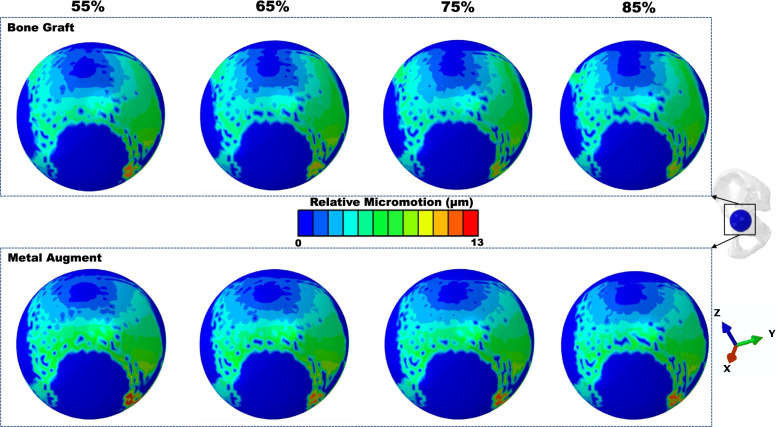
Relative micromotion distribution of cup-host bone interface in different models

The peak relative micromotion between shell and host bone (including BG/MA contact area) was higher in the MA situation (12.61 vs 11.13 µm) (Fig. 2b). The higher relative micromotion was in zone 3, for either BG (11.13 µm) or MA (12.61 µm) situation (Fig. 3b). The concentration of relative micromotion for the shell was present in the inferoposterior corner of zone 3 for either BG or MA situation (Fig. 6).

**Fig. 7:**
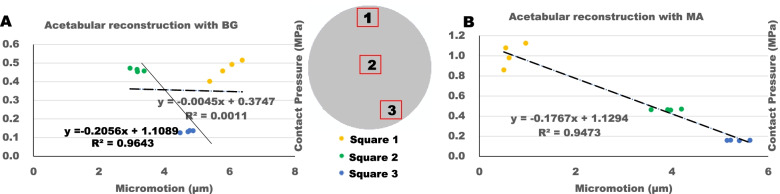
The correlation of contact pressure and micromotion in cup-host bone (including BG/MA) interface of different models. **a** Acetabular reconstruction with BG models. **b** Acetabular reconstruction with MA models

The relationship of contact stress and micromotion was compared between the two reconstructed materials (Fig. 7). Three regions (3 × 3 mm squares) inside each of the DeLee and Charnley Zones were harvested. Averaged contact pressure and micromotion in each square of every model were used to represent the contact pressure and micromotion in that region. From the results of linear regression analysis, the contact pressure and micromotion had a negative relationship. However, the acetabular reconstruction with BG had a poor fitness with the R^2^ value of 0.001 (Fig. 7A) compared to the MA situation with the R^2^ value of 0.947 (Fig. 7B).

## Discussion

The primary purpose of the study was to quantitatively compare the influence of two materials (Ti6Al4V augment and autologous bone graft) for acetabular reconstruction of DDH on acetabular stability after THA using a finite element method. Crown II and III FE models were simulated by elevating the femoral head center. The mechanical parameters of contact pressure predicted with CPRESS, relative micromotion calculated with CSLIP in the interface between shell and host bone were utilized to evaluate the acetabular stability.

The overall peak contact pressure was slightly higher in the MA situation, indicating that acetabular reconstruction with MA such as Titanium alloy had more contact pressure compared to BG such as structural bone graft (Fig. 2a). However, the magnitude was higher than the normal hip peak contact pressure, even the dysplasia hip peak contact pressure in the single-leg standing condition [40–42]. The reason may be the biomechanics of hip had been changed by THA compared to the normal configuration [43, 44], including the effect of fraction, material properties and porosity between the interface of implant-bone [45]. It has been confirmed that acetabular component with porosity was able to reduce the maximum contact stress on the bone surface [46].

The peak contact pressure decreased in Zone 2 of the DeLee and Charnley Zones either for the BG models or the MA models compared with Zone 1, 3 (Fig. 3a), which was corresponded with the contact stress distribution (Figs. 4 and 5). The interface stress transmission from acetabular component to surrounding bone featured in the superior dome of Zone 1, and inferioanterior area of pubic branch. However, the load transfer path was different in Zone 1, for BG models, the high local contact stresses were found at the junction of BG and host bone while for MA models the concentrated contact stresses were at the surface of MA. The results were similar with the biomechanical study [47] about segmental acetabular rim defects reconstructed with bone graft and reinforcement ring, that the peak stress concentration was located in the superior-posterior of the acetabulum. This suggested that the full fixation postoperatively in the superior-posterior dome should be needed for the initial stability.

The peak micromotion was slightly higher for the MA models (11.92–12.61 µm) (Fig. 2b), lower than the relative displacement (20–40 µm) for adequate bone ingrowth from reports [48, 49], indicating that acetabular reconstruction with MA and BG could provide enough initial stability for cup bone ingrowth to guarantee good long-term results. The predicted relative micromotion between the interface of cup and host bone was consistent with the previous biomechanical study [50]. However, the acetabular reconstruction with GB has the disadvantage with graft resorption and collapse at the early postoperative stage [51–53]. Alternatively, the metal augments such as Tritanium acetabular wedge augments can be used with less micromotion for adequate bone ingrowth and stable clinical follow-up results [54, 55].

Compared to BG models in DeLee and Charnley zones, the peak micromotion decreased in zone 1 of MA models without zone 2 and 3 (Fig. 3b), indicating that acetabular reconstruction with MA had an excessive direct fixation with the cup (zone1), but the MA and BG materials had little influence on the host bone (zone 2, 3). The results were corresponded with the interface micromotion distribution of cup and host bone (Fig. 6), the micromotion distribution was similar in zone 2 and 3. High micromotion was located in the inferioposterior corner of ischial branch and inferioanterior corner of pubic branch. Numerical results indicated that support from superior dome, ischial branch and pubic branch was necessary to obtain the initial stability in case of DDH or revision THA [56, 57].

The micromotion and contact stress had a negative relationship in bone-implant surface including the implant-augment contact area. For MA models, a good fitness with *R*^*2*^ = 0.947 was present, because the less micromotion was, the more contact stress displaced in Square 1 of implant-MA surface. In contrast, for BG models, the more micromotion was, the higher contact stress in Square 1 of implant-BG surface compared to the implant-MA surface, however, the good fitness with *R*^*2*^ = 0.964 was present only considering the implant-host bone surface (Fig. 7A black solid line). This indicated that the MA was able to provide stronger direct fixation with cup connection.

Limitations were: (1) The present study was performed with a computational simulation method, though it was validated [24, 58, 59]. The biomechanical test should be added to enrich the results more convincingly [60, 61]. (2) There are many factors that influence the secondary bone fixation or the cup stability [62], the main factor was the type of implant surface coated design [48]. The cup and MA used in the study was not the porous coated design, this may influence the predicted results, however, the fraction parameters between contact surface were defined as porous coated situation [16, 22]. (3) The acetabular cup fixation method was not the press-fit technique used in clinical [31], but a press-fit contact pattern between cup and bone was decided by simulating an equivalent friction coefficient from literature [16], and the cup-bone relative micromotion may be changed [32]. (4) The present study was only focus on acetabular component-host bone interface to evaluate the stability of cup, the augment-bone interface should be investigated further to study the biomechanical behavior of MA and BG directly. (5) There was only one example of the FEA model, which may affect the universality of the study, and it was tested mechanically without muscle force, just with the contact hip joint force instead, which should be considered in the future research. (6) theoretically, the BG should be priority, because of its synostosis with host bone [63], while the MA with host bone was an osteointegration [64], however, the biological factors was not considered in present study, just the mechanical properties addressed.

## Conclusions

Acetabular reconstruction of DDH with MA in THA is an emerging technique compared to BG with a long history. From the predicted results, the load transfer path was different in the implant-bone interface with the two augment materials. And a higher micromotion was found in the MA models, however, the micromotions of the both in the implant-bone interface were lower than measurements for adequate bone ingrowth, especially, MA -implant interface had a less micromotion than the GB-implant interface. Thus, we recommended the MA can be used as an option, even for Crowe III, however, the decision should be made from clinical follow-up results.

## Data Availability

The data that support the findings of this study are available from CP, but restrictions apply to the availability of these data, which were used under license for the current study, and so are not publicly available. Data are however available from the authors upon reasonable request and with permission of CP.

## Abbreviations

THA: Total Hip Arthroplasty
DDH: Adult Developmental Dysplasia of the Hip
HCOR: Hip Center of Rotation (COR)
QCT: Quantitative Computed Tomography
CAD: Computer Assisted Design
MA: Metal Augment
BG: Bone Graft
APP: Anterior Pelvic Plane
HU: Hounsfield Unit
FEA: Finite Element Analysis
